# Polycyclic aromatic hydrocarbon (PAH) biodegradation capacity revealed by a genome-function relationship approach

**DOI:** 10.1186/s40793-023-00497-7

**Published:** 2023-04-30

**Authors:** Yue Huang, Liguan Li, Xiaole Yin, Tong Zhang

**Affiliations:** Environmental Microbiome Engineering and Biotechnology Lab, Department of Civil Engineering, The University of Hong Kong, Pokfulam Road, Hong Kong, China

**Keywords:** PAH, Biodegradation, Database mining, Functional gene, Genome-centric analysis, Genotype–phenotype relationship, Random forest

## Abstract

**Background:**

Polycyclic aromatic hydrocarbon (PAH) contamination has been a worldwide environmental issue because of its impact on ecosystems and human health. Biodegradation plays an important role in PAH removal in natural environments. To date, many PAH-degrading strains and degradation genes have been reported. However, a comprehensive PAH-degrading gene database is still lacking, hindering a deep understanding of PAH degraders in the era of big data. Furthermore, the relationships between the PAH-catabolic genotype and phenotype remain unclear.

**Results:**

Here, we established a bacterial PAH-degrading gene database and explored PAH biodegradation capability via a genome-function relationship approach. The investigation of functional genes in the experimentally verified PAH degraders indicated that genes encoding hydratase-aldolase could serve as a biomarker for preliminarily identifying potential degraders. Additionally, a genome-centric interpretation of PAH-degrading genes was performed in the public genome database, demonstrating that they were ubiquitous in *Proteobacteria* and *Actinobacteria*. Meanwhile, the global phylogenetic distribution was generally consistent with the culture-based evidence. Notably, a few strains affiliated with the genera without any previously known PAH degraders (*Hyphomonas*, *Hoeflea*, *Henriciella*, *Saccharomonospora*, *Sciscionella*, *Tepidiphilus*, and *Xenophilus*) also bore a complete PAH-catabolic gene cluster, implying their potential of PAH biodegradation. Moreover, a random forest analysis was applied to predict the PAH-degrading trait in the complete genome database, revealing 28 newly predicted PAH degraders, of which nine strains encoded a complete PAH-catabolic pathway.

**Conclusions:**

Our results established a comprehensive PAH-degrading gene database and a genome-function relationship approach, which revealed several potential novel PAH-degrader lineages. Importantly, this genome-centric and function-oriented approach can overcome the bottleneck of conventional cultivation-based biodegradation research and substantially expand our current knowledge on the potential degraders of environmental pollutants.

**Supplementary Information:**

The online version contains supplementary material available at 10.1186/s40793-023-00497-7.

## Background

Polycyclic aromatic hydrocarbon (PAH) contamination has been a global environmental issue for decades. PAHs are a group of organic compounds composed of two or more fused aromatic rings with natural and anthropogenic sources [1–3], which are well-recognized as carcinogenic, teratogenic, and genotoxic compounds [4–6]. They are ubiquitous in the environments [3, 7, 8] at relatively high concentrations, likely to accumulate in animal tissues and vegetation due to their high lipophilicity [9–11], and thus harmful to ecosystems and human health. Although PAHs could be eliminated by adsorption [12], volatilization, and photochemical degradation [13], microbial degradation is one of the dominant mechanisms of PAH removal in natural environments [7, 14, 15]. In the past half-century, a wide variety of PAH-catabolic bacteria, archaea, fungi, and microalgae have been isolated mostly from contaminated soils and sediments [16], among which bacteria-mediated biodegradation has been extensively studied. Currently, the identified PAH-degrading bacteria are distributed in diverse genera, such as *Pseudomonas* spp. [17, 18], *Sphingomonas* spp. [19, 20], *Mycobacterium* spp. [21, 22], *Rhodococcus* spp. [23, 24], *Burkholderia* spp. [25, 26], etc. Nevertheless, it is believed that most PAH-degrading bacteria still hide in plain sight due to the isolation bottleneck [27]. Therefore, there is a dire need for a new method to efficiently identify novel potential PAH degraders.

Basically, biological traits are developed based on their encoding genes. Traditional culture-based approaches have set a good foundation for understanding PAH biodegradation pathways, functional genes, and enzyme-catalyzed reactions [15]. Commonly, in the upper pathway, biodegradation of PAHs is initially catalyzed by ring-hydroxylating dioxygenases (RHDs) [28] and followed by other enzymes encoded by *nah* gene cluster (*nahBCDEF*), which is well characterized in naphthalene and phenanthrene aerobic biodegradation [29, 30], but with a broad substrate specificity to aromatic compounds [31]. Conventionally, the *nahAc* encoding α-subunit of RHDs was usually employed as the biomarker to demonstrate the diversity and abundance of RHDs in PAH-degrading isolates and multiplex systems by quantitative real-time PCR [32, 33]. Nevertheless, owing to its high specificity, the primers of *nahAc* only target a relatively narrow range of *nahAc*-like sequences and result in an under-estimated PAH-degrading consortia [34, 35]. Moreover, other PAH-catabolic gene clusters also exist in Gram-negative bacteria, including *nag* [36], *pah* [18], *ndo* [37], and *phn* [25] gene clusters, as well as in Gram-positive bacteria, including *nar* [16, 38], *phd* [39], *nid* [40], and *pdo* [41] gene clusters. However, a unified database integrating the diverse PAH-degrading genes for exploring potential novel PAH degraders is still lacking.

In the era of high-throughput sequencing, access to the genome information of currently uncultivable microbes has opened a new window to explore this topic. Concurrent with the advance of long-read sequencing technologies, the number of high-quality genomes increased exponentially. The current technology improvements make it possible to interpret biological traits based on their whole genomes instead of single or multiple biomarkers. Meanwhile, the biodegradation processes of PAHs and functional enzymes are very diverse and complicated in different species (i.e., *Pseudomonas* spp., *Mycobacterium* spp., and *Rhodococcus* spp.) and habitats (i.e., aerobic, anoxic, and anaerobic) [16, 42]. The unknown alternative genes or enzymes for individual steps may generally exist, which are not represented in the currently available gene database. Hence, it remains a big challenge to properly identify these genetic hints based on similarity search, not to mention their functional potentials. Fortunately, the introduction of the Hidden Markov Model (HMM) [43] has made it possible to detect remote homology between proteins with high efficiency and accuracy. This is a popular method predicting biological functions based on conserved protein domains, and has been widely applied to gene identification [44], phylogenetic analysis [45], and database construction [46]. Therefore, HMM was employed in the present study aiming to improve the accuracy of functional gene identification and profile the functional genes at scales ranging from a single isolate to the whole genome database.

Herein, we collected the protein sequences of key enzymes responsible for the upper pathway of PAH metabolism and established a dedicated database for similarity- and HMM-based searches. By investigating the distribution of PAH-degrading genes in the known degraders with complete genomes, we evaluated the performance of two alignment methods, paving the way for the identification of novel PAH degraders on a large scale. Then, a genome-centric interpretation of PAH-catabolic genes was conducted in the NCBI genome database, aiming to depict a phylogenetic distribution of PAH-degrading genes and discover novel lineages containing potential PAH degraders. Finally, a preliminary exploration to link the PAH-catabolic genotypes to phenotypes via a random forest analysis was performed and applied to predict the PAH biodegradation trait in the complete genome database. In general, this study based on the genome-function relationship represents a paradigm shift and provides a novel insight into conventional biodegradation research.

## Methods

### Construction and validation of PAH-degrading gene database

**Fig. 1:**
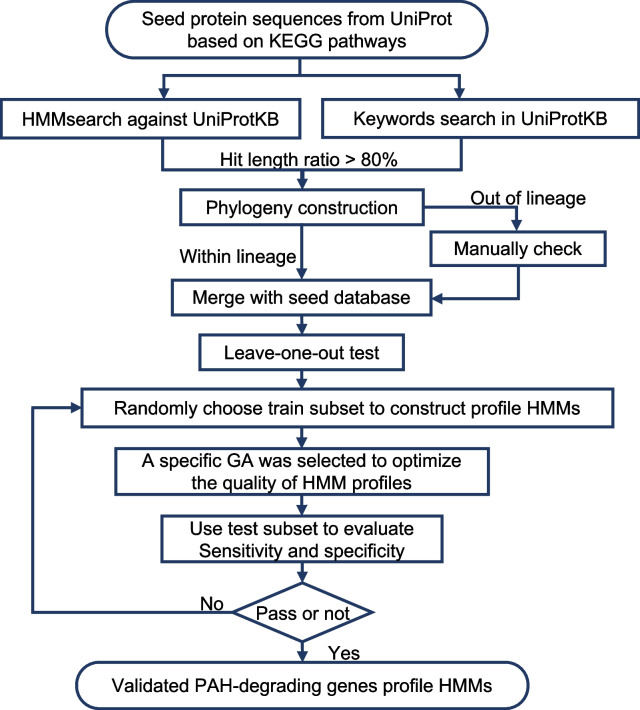
The workflow of PAH-degrading gene database construction

The PAH-degrading gene database was constructed following the workflow depicted in Fig. 1. The seed protein sequences were collected based on naphthalene and phenanthrene aerobic biodegradation pathways in the Kyoto Encyclopedia of Genes and Genomes (KEGG) pathway database. Those PAH-degrading genes with more than three protein sequences were used to construct initial profile HMMs. To retrieve more PAH-degrading protein sequences, a database search against the UniProtKB was performed by two strategies, namely HMMsearch and keywords. Then, a phylogenetic tree topology was constructed using MEGAX (v10.0.5) [47], and the questionable sequence, which was distinct from the core cluster, was filtered out after manually checking. Subsequently, a leave-one-out test was applied for fine adjustment of the protein database, where the sequence leading to significantly low sensitivity and specificity values was marked. Next, a training subset (two-thirds of sequences) was randomly chosen from the enriched database to construct profile HMM, while the rest were used as the test dataset for validation. A specific gathering threshold (GA) was selected for each profile HMM, and the optimal GA value was obtained according to the sensitivity and specificity using a bash script. The profile HMMs with both sensitivity and specificity values exceeding 90% were retained as the validated models. It is a loop that will stop when there is no further addition to this expanded database to form the final version.

### Genome and protein sequence

The GenBank flat file (.gbff) of 22,507 bacteria with complete genomes, 263,643 bacteria with draft genomes (updated on March 2021), and 7045 archaeal genomes (updated on Apr 2023) were downloaded from the NCBI database. Their nucleotide sequences were extracted by an in-house Python script. Then, their open reading frames (ORFs) were predicted using prodigal (v2.6.3) [48].

### PAH-degrading genes identification and PAH-degrading bacteria prediction

Two different methods (similarity- and HMM-based pipelines) were adapted to identify PAH-degrading genes in 47 experimentally confirmed PAH degraders with complete genomes. The similarity-based search was performed using DIAMOND (v2.0.8.146) [49] with an identity of over 70% and a hit length ratio of over 70%. For the HMM-based search, MAFFT (v7.310) [50] and hmmbuild from HMMER 3.0 suite [51] were used to align sequences and generate the profile Hidden Markov models. PAH-catabolic genes were identified using the profile HMMs and hmmsearch at -cut_ga mode. After comparing the accuracy and efficiency of the two methods, only the HMM-based approach was employed to interpret the distribution of PAH-degrading genes in the public genome database. The phylogenetic trees were visualized using iTOL (v6.6) [52].

To further investigate the genotype–phenotype relationships in the PAH-degrading bacteria, a supervised learning algorithm, Random Forest, was applied in this study. The analysis was performed with the R package ‘randomForest’ [53] using the maximum GA bit score of each gene in the genome. In addition to the numbers of variables at each node (m_try_) and trees in the forest (n_tree_), the ratio of True/False in the training dataset was also considered since the majority of bacteria were not PAH degraders. Basically, two-thirds of genomes were randomly chosen from the dataset for training the model, while the rest were utilized for verification. Four standard metrics are used to evaluate the quality of the proposed model, consisting of sensitivity (Sn), specificity (Sp), overall accuracy (Acc), and Mathew’s correlation coefficient (MCC) with the following definitions:

$$Sn = \frac{TP}{{TP + FN}}$$

$$Sp = \frac{TN}{{TN + FP}}$$

$$Acc = \frac{TP + TN}{{TP + TN + FP + FN}}$$

$$MCC = \frac{TP \times TN - FP \times FN}{{\sqrt {\left( {TP + FP} \right) \times \left( {TN + FN} \right) \times \left( {TP + FN} \right) \times \left( {TN + FP} \right)} }}$$

where *TP* (true positive) and *TN* (true negative) are correctly predicted PAH-degrading positive and negative bacteria, respectively. *FP* (false positive) and *FN* (false negative) indicate falsely predicted PAH-degrading positive and negative bacteria, respectively. Among these metrics, *MCC* is the most stringent one, as it takes into account both accuracy and error rates.

## Results and discussion

### Experimentally verified PAH-degrading strains

**Fig. 2:**
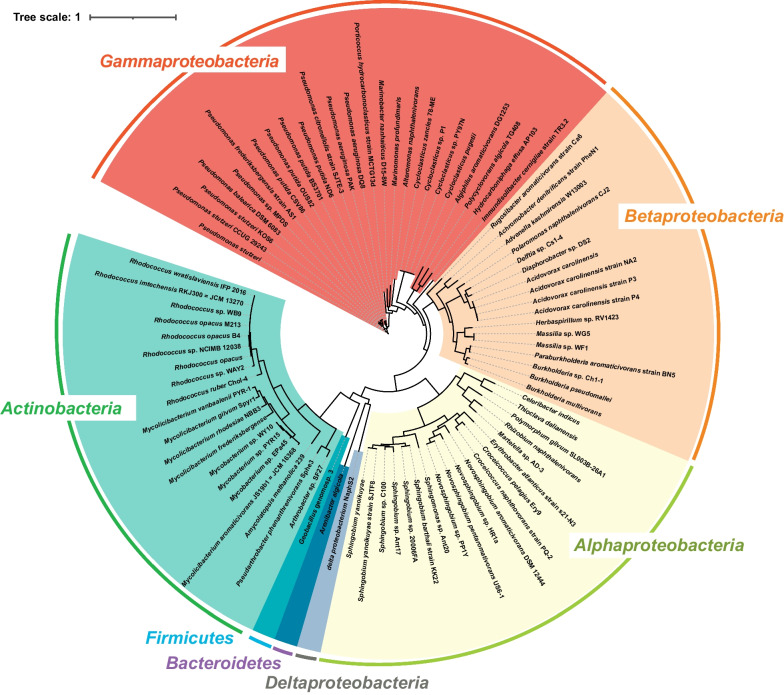
A phylogenetic tree of the reported PAH-degrading bacterial strains based on their genomes. The tree was constructed using gtdb-tk (v1.7.1) [91]. The details of these PAH-degrading strains are summarized in Additional file 1: Table S1

To date, numerous PAH-degrading strains (more than 200) have been isolated from various habitats as aforementioned. More than 95% of them were from the domain of bacteria, but limited information was available regarding their genome. Therefore, the PAH-degrading strain database only comprised genome sequences of 95 reported PAH-degrading bacterial strains. The detailed information was summarized in the supplementary material (Additional file 1: Table S1). The profiling of these known PAH-degrading strains (Fig. 2) demonstrated that they were phylogenetically diverse owing to the evolutions in different habitats as well as horizontal gene transfer [54, 55]. Notably, 95% of those degradation strains were affiliated with two phyla of *Proteobacteria* and *Actinobacteria*. Of the 42 genera represented, *Pseudomonas* (14%) constitutes most of the reported PAH-degrading strains, followed by *Rhodococcus* (10%), *Mycobacterium* (9%), and *Sphingobium* (7%). Additionally, most of the identified PAH-degrading strains could catabolite multiple PAHs, such as *Pseudomonas putida* OUS82 and *Mycolicibacterium vanbaalenii* PYR-1, supporting that the functional enzymes have a broad substrate specificity to multiple aromatic compounds (Additional file 1: Table S1).

### PAH-degrading protein sequence database

In addition to the PAH-degrading strain database, it is critical to construct a protein database for the annotation of related gene clusters. Enzymes related to the aerobic biodegradation pathways of six common PAHs (naphthalene, phenanthrene, anthracene, fluorene, pyrene, and benzo[*a*]pyrene) have been well archived in the KEGG database, as well as their associated enzymes. The protein sequences were retrieved based on the naphthalene and phenanthrene biodegradation pathways to form a seed database. A comprehensive database was prepared by expanding the seed database following the workflow described in the Methods. After expanding, the *nar* gene cluster was included to improve the sensitivity for Gram-positive bacteria, such as *Rhodococcus* spp. Simultaneously, for RHDs, only *nahAc* genes encoding the ion-sulfur subunit were retained in the database because they were conserved and could serve as a biomarker for RHDs [32, 56, 57]. Likewise, *nahF* and *phdK* genes were excluded due to their poor phylogenetic conservation, which is hard to choose a suitable GA cut-off to ensure both sensitivity and specificity. Notably, *nag*, *ndo*, *pah*, *phn*, *dox*, and *bph* gene clusters were also included in the current database since they were homologous to the *nah* genes cluster. Eventually, a total of 1,191 manually checked PAH-degrading protein sequences were included in the comprehensive database, a twice-fold increase in the number of sequences compared with the seed database (Additional file 3: Figure S1). These reference sequences were from 17 different degradation genes, which could be classified into three types based on degradation mechanisms, namely (1) *nah*, (2) *nid* and *phd*, and (3) *nar* gene clusters (Additional file 3: Figures S2–4). The detailed information on each protein sequence was summarized in the Supplementary Information (Additional file 2: Tables S2–8). In the database, the *nah* gene cluster (843 protein sequences, 71% of total sequences in the database) was the most dominant type, followed by the *nid* and *phd* gene cluster (26%) and the *nar* gene cluster (3%).

### Similarity- and HMM-based searches for verified PAH-degrading strains

**Fig. 3:**
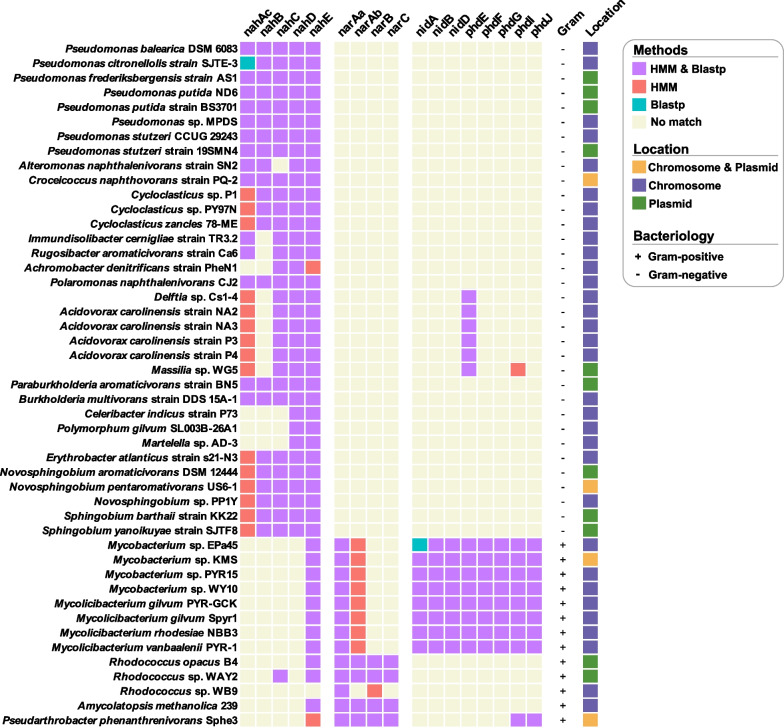
The PAH-degrading gene distribution in 47 experimentally validated PAH-degrading bacteria. The heatmap compares similarity- and HMM-based searches. The right column demonstrates the location of PAH-degrading genes, and detailed information is depicted in Additional file 3: Figure S6. Notably, the identifications of *nahE* in *Mycobacteriaceae* and *Rhodococcus* were *phdJ* and *narC*, respectively

In the present study, similarity- and HMM-based methods were employed to identify functional genes in the 47 experimentally verified PAH-degrading strains (NCBI assembly level = Complete) (Additional file 1: Table S1 and Fig. 3). Both approaches could accurately identify most PAH-degrading genes, whereas the HMM-based method allows us to retrieve the potential PAH-degrading genes which, however, cannot be identified by the similarity-based strategy, such as *nahAc* genes in *Cycloclasticus* and *Acidovorax carolinensis*. Because HMM captures conserved protein domains necessary for the protein function, the HMM-based method thus is more sensitive and rapid in detecting remotely homologous sequences on a large scale. Notably, not all the PAH-degrading strains have a complete pathway of PAH biodegradation, such as *Archomobacter denitrificans* PheN1, *Celeribacter indicus* P73, and *Martelella* sp. AD-3 (Fig. 3). A parsimonious interpretation of gene deletion was the existence of alternative genes/enzymes for the individual steps in these strains, which have not yet been reported.

Furthermore, both methods can accurately distinguish the *nahAc*, *nidA*, and *narAa* encoding the large subunits of RHDs even though they showed significant but moderate sequence homology to each other [21, 34]. However, both approaches cannot alleviate the misclassification issue on *nahE*, *phdJ*, and *narC* in Gram-positive strains. The misclassification was defined as the single protein sequence being classified into multiple gene types under the optimized cut-off parameters. For example, the *phdJ* gene was identified as the *nahE* gene by similarity- and HMM-based methods in *Mycobacteriaceae* spp. Because both NahE and PhdJ were *N*-acetylneuraminate lyase subgroup members with a conserved (β/α)_8_ barrel structure, two strictly conserved active site residues (tyrosine and lysine), and a GXXGE motif (Gly-61, Thr-62, Phe-63, Gly-64, and Glu-65) [58]. It is not easy to distinguish them based on either similarity of the whole sequence or protein domains. In addition, the *narC* gene encoding aldolase in *Rhodococcus* spp. was classified into the *nahE* group, which was located near the *narB* gene and involved in the biodegradation of PAH compounds [24, 38, 59]. The misclassification suggested that hydratase-aldolase-coding genes were more conservative than RHDs-coding genes, consistent with the phylogenetic analysis of these PAH-degrading genes (Additional file 3: Figure S2). Meanwhile, hydratase-aldolase-coding genes were identified in 46 strains (98%), and, therefore, genes encoding hydratase-aldolase may be a superior biomarker for PAH degraders. Moreover, the primers to amplify *nahE*, *phdJ*, or *narC* have been well designed and evaluated in the previous studies [34, 58], providing a rapid way to initially explore the ecological role and degradation potential of PAH-catabolic bacteria in the natural environment. Notably, the conclusion from genome-centric interpretation was in agreement with the results based on the phylogenetic analysis of PAH-catabolic enzymes, which proposed *pahE* (including *nahE*, *phdJ*, and *narC*) as a functional marker because all the enzymes encoded by *pahE* clustered in an independent clade [34].

Intriguingly, *narAa* and *narAb* were also identified in *Mycobacteriaceae* spp., which were Gram-positive strains and contained a complete *nid* and *phd* gene cluster as well. We suspected the involvement of the enzyme encoded by *narA* during the initial attack of PAH biodegradation, but scientific evidence is still lacking. It requires more experimental validation conducted by transcriptomics to further investigate the expression of *narA* during the biodegradation process. Meanwhile, the location and gene arrangement of PAH-catabolic genes were investigated in the 47 identified PAH-degrading strains, showing similar gene arrangements in *Pseudomonadaceae* spp*., Comamonadaceae* spp., and *Mycobacteriaceae* spp. (Additional file 3: Figure S5). Interestingly, plasmid-bearing catabolic genes were detected in *Pseudomonas* spp., *Sphingobium* spp., and *Rhodococcus* spp. with a high frequency (8 out of 13 strains) (Fig. 3 and S6). This result was consistent with numerous studies that characterized the functional genes in PAH degraders in the genera of *Pseudomonas* [54, 60], *Sphingobium* [61, 62], and *Rhodococcus* [24, 59]. The presence of degradation genes on the transmissible plasmids, such as NAH7 [63], pKS14 [64], and pNL1 [65], has indicated easy spreading of PAH-catabolic ability via horizontal gene transfer in contaminated sites [54, 66].

### The distribution of PAH-catabolic genes and strains in the public database

**Fig. 4:**
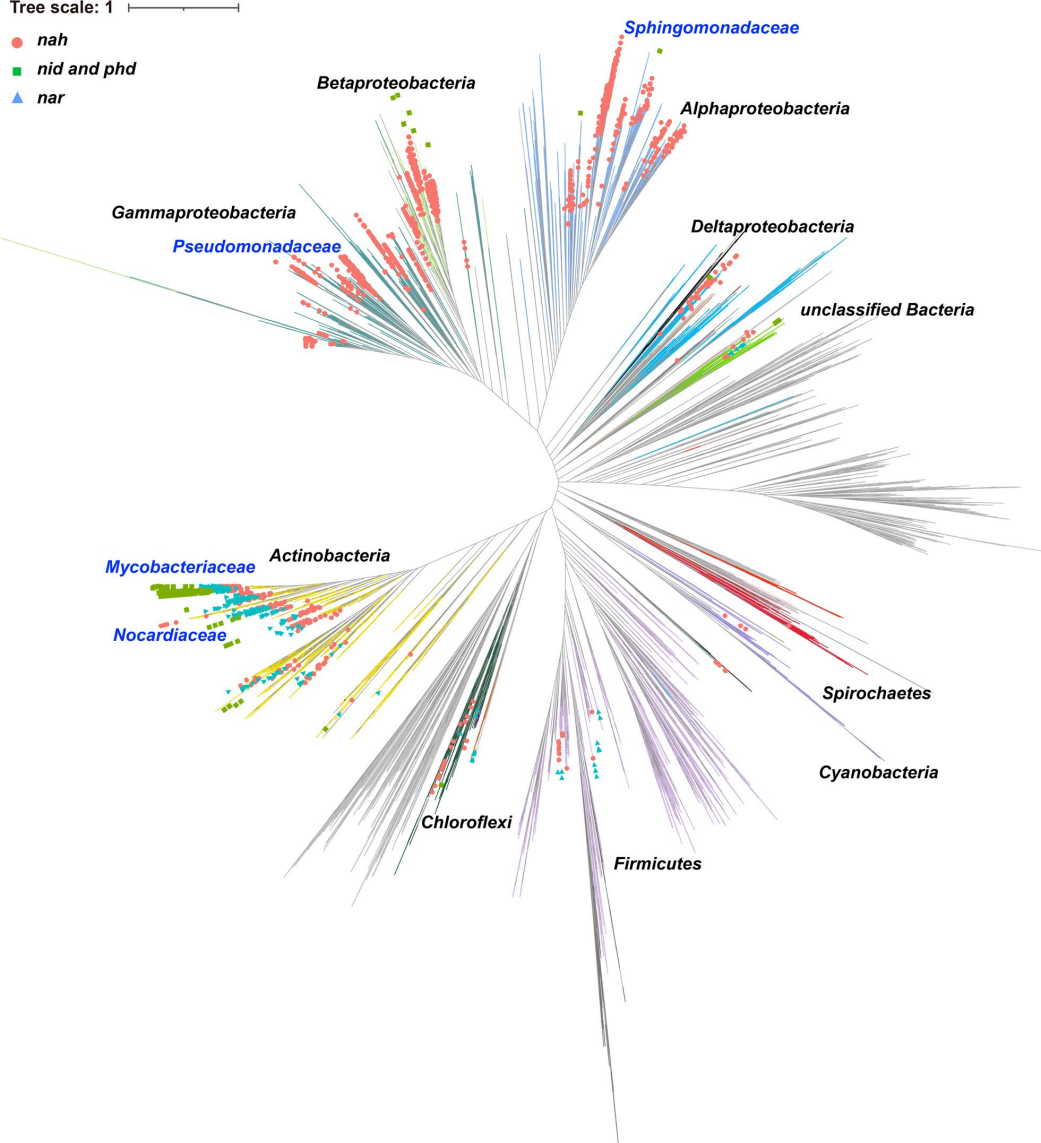
The phylogenetic distribution of PAH-degrading genes. *nah* genes (*nahAc*, *nahB*, *nahC*, *nahD*, and *nahE*) are shown in pink circle plots. *nar* genes (*narAa*, *narAb*, *narB,* and *narC*) are shown in blue triangle plots. *nid* and *phd* genes (*nidA*, *nidB*, *nidD*, *phdE*, *phdF*, *phdG*, *phdI,* and *phdJ*) are shown in green square plots. The top four families with the most PAH-catabolic genes, *Pseudomonadaceae*, *Sphingomonadaceae*, *Mycobacteriaceae*, and *Nocardiaceae*, are highlighted in blue color

Subsequently, a large-scale survey in the NCBI database, including all complete-, scaffold-, contig-, and chromosome-level bacterial assemblies, was conducted to investigate the genome-centric portrait of PAH-degrading genes (Fig. 4). At the phylum level, the PAH-degrading genes were ubiquitous in *Proteobacteria* and *Actinobacteria*, in agreement with the result of our collected PAH-degrading strain database, proving the representativeness of our genomic database. Meanwhile, they were also found in other phyla, such as *Firmicutes*, and *Chloroflexi*, implying a phylogenetic diversity of PAH-degrading strains. Significantly, *nah* genes were the most widely distributed degradation genes in the public database, especially in Gram-negative strains of *Proteobacteria*. In *Actinobacteria*, three types of PAH-catabolic genes were observed, where the enzymes encoded by *nid* and *phd* genes were a conservative trait for PAH-catabolic strains in the family of *Mycobacteriaceae*. Moreover, the enzymes encoded by *nar* gene cluster were only identified in Gram-positive strains. At the family level, over 30% of strains with PAH-degrading genes were affiliated with *Sphingomonadaceae*, *Pseudomonadaceae*, *Nocardiaceae*, and *Mycobacteriaceae*, indicating that these strains constituted the majority of PAH degraders. Generally, these genome-centric results were consistent with cultivation-based experimental data (Fig. 2).

Additionally, there were 173 strains with a complete PAH-catabolic gene cluster and 52 with a near-complete PAH-catabolic gene cluster (one gene missing). The phylogenetic tree and details of these 225 strains were summarized in Additional file 3: Figure S7 and Additional file 2: Table S19, respectively. Intriguingly, in addition to those strains phylogenetically close to the well-known PAH degraders, nine strains in the genera of *Hyphomonas*, *Hoeflea*, *Henriciella*, *Saccharomonospora*, *Sciscionella*, *Tepidiphilus*, and *Xenophilus* also bore the potential of PAH biodegradation owing to their possession of a complete PHA-degrading gene cluster (Additional file 3: Figure S8). Seven of them were isolated from marine water, and the rest two were from production water (*Tepidiphilus* sp. J18 [67]) and soil (*Xenophilus azovorans* DSM 13,620 [68]). Despite the enrichment of these genera observed in PAH-contaminated sites [69, 70], related PAH-degrading isolates have not yet been reported, probably owing to the isolation bottleneck. Therefore, these seven genera were potential novel PAH-degrader lineages.

Furthermore, we performed a preliminary investigation in archaeal assemblies (data not shown), revealing that two halophilic archaea, namely *Halopenitus malekzadehii* and *Halobellus rufus*, contained a *nahE* gene. They were affiliated with *Halorubraceae* and *Haloferacaceae*, respectively, and phylogenetically related to the identified PAH-degrading archaea (at the family level) [71, 72]. Interestingly, a gentisate-1,2-dioxygenase-like gene (*gdoA*) was also identified in both archaeal assemblies based on a similarity search (> 75% identity), which was homologous to bacterial dioxygenases and involved in the aromatic degradation in *Haloferacaceae* sp [73]. Nevertheless, the exploration of archaea-mediated PAH biodegradation is still in its infancy, and archaeal PAH-degrading genes were not included in the present PAH-degrading gene database, requiring more studies to pave the way for investigating biological traits on a genome scale.

### Prediction of PAH-degrading strains in the complete genome database

In the random forest algorithm, the three most important parameters were the number of trees (n_tree_), variables randomly chosen at each node split (m_try_), and the composition of the training dataset. When the number of non-degraders was 3 to 8 folds larger than the number of PAH degraders in the dataset, the classifiers achieved a high accuracy with average *MCC* values of ~ 0.982 (Additional file 3: Figure S8a). Theoretically, a higher value of n_tree_ will lead to better accuracy, but the computation time will increase simultaneously. Additionally, theoretical and empirical research has highlighted that classification accuracy is more sensitive to m_try_ than n_tree_ [74]. Therefore, n_tree_ was fixed at 2000 in the present study, and m_try_ was optimized from 1 to 17 with a step size of 1 to generate the prediction model. The error rate decreased with the increase of m_try_ value and leveled off at a low error rate of 0.056 after the m_try_ value was set as 3 (Additional file 3: Figure S8c). Then, under the optimized parameters, we noticed that *nahE*, *phdJ*, and *phdG* genes played a crucial role in the classifier based on the high mean decrease accuracy and mean decrease gini values [75, 76], supporting the proposal of genes encoding hydratase-aldolase as a new biomarker for PAH-degrading strains [34] (Additional file 3: Figure S9). In contrast, *nahF* with low mean decrease accuracy and gini values was, therefore, excluded from the PAH-degrading gene database.

**Fig. 5:**
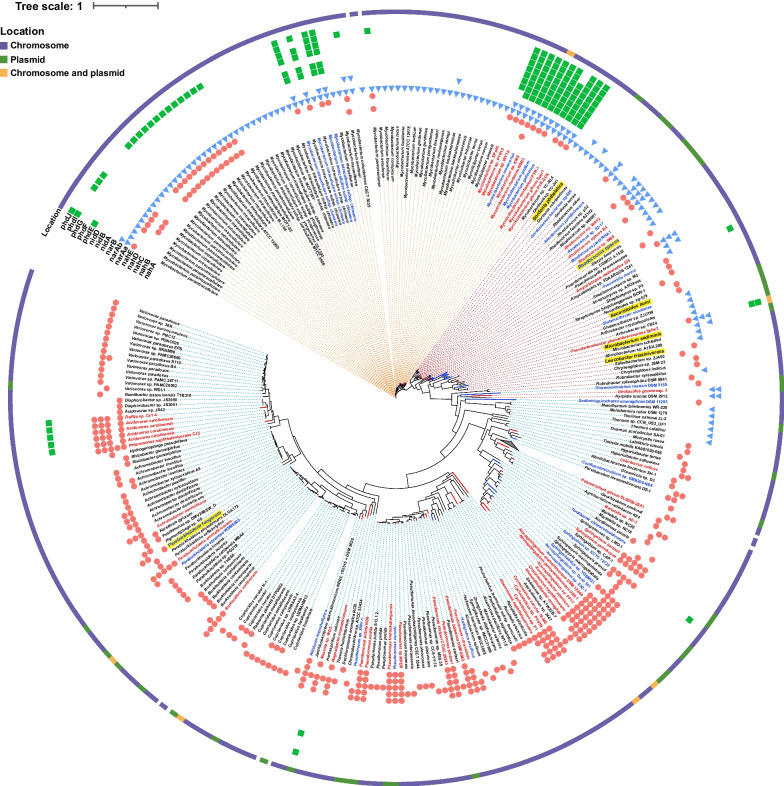
The distribution of PAH-degrading genes in the NCBI complete genome database. From the outside to the inside: (1) The color in the circle indicates the location of identified PAH-degrading genes; (2) The pink circle, blue triangle, and green square indicate *nah*, *nar*, and *nid* and *phd* gene cluster, respectively. (3) The red tag represents the 47 identified PAH-degrading bacteria, while the blue tag denotes the 28 predicted PAH-degrading bacteria by random forest analysis. Six pure strains selected for experimental verification are depicted with yellow shades. (4) The species affiliated with the family *Mycobacteriaceae* are distinguished by orange branch dashed lines, whereas the branch dashed lines of Gram-negative and Gram-positive strains are blue and red, respectively. Notably, the *nahE* identified in *Mycobacteriaceae* was misclassification

The optimized model (random forest classifier) was applied to predict PAH-degrading bacterial strains based on the results from the HMM-based search in the complete genome database (Fig. 5). Given that the degradation mechanism in *Mycobacteriaceae* was only reported via the enzymes encoded by *nid* and *phd* genes, strains were divided into three groups before model construction and prediction, namely, Gram-negative, Gram-positive, and *Mycobacteriaceae*. Only the strain whose prediction was consistent with its group tag would be output. In total, 28 strains were newly predicted to be capable of PAH biodegradation, including 14 strains from the Gram-negative group, 7 strains from the Gram-positive group, and 7 strains from the *Mycobacteriaceae* group. Among these newly predicted PAH degraders, nine strains contained a complete PAH-catabolic gene cluster. In the Gram-negative group, most strains (12) were from *Proteobacteria* and phylogenetically related to the identified degraders. In addition, *Thermomicrobium roseum* DSM 5159 and *Sediminispirochaeta smaragdinae* DSM 11,293 only harbored the *nahE* gene and were affiliated with the phyla of *Chloroflexi* and *Spirochaetes*, respectively. However, no PAH-degrading strain has been isolated from these two phyla to date, and the prediction needs further evaluation. Probably, this hydratase-aldolase-coding gene was involved in other catabolic processes since both strains were isolated from oligotrophic environments [77, 78]. In the Gram-positive group, all potential PAH-degrading strains were affiliated with *Actinobacteria*, of which PAH degrader has been reported previously.

Apart from the nine strains with a complete PAH-degrading pathway which were not publicly available, we purchased six strains from DSMZ for further experimental verification, including two predicted PAH-catabolic strains. However, none of them showed a PAH biodegradation capability during 30-day incubation with naphthalene as the sole carbon source (Additional file 3: Figure S10, the degradation experiment was described in SI). But gene expression is an intricate process controlled by the joint effect of multiple aspects, such as environmental factors [79], quorum sensing [80], etc. In addition, promoters (e.g., P_*pahA*_ and P_*pahR*_ [81]) and regulators (e.g., *nahR* [82], *nagR* [83], and *narR* [59]) also play an essential role in the expression of PAH degradation. Moreover, the expression of regulatory genes requires induction of naphthalene [59, 84] or its degradation metabolite, salicylate [29, 85]. Hence, we could only reach a limited conclusion that these strains did not exhibit their PAH-catabolic trait under this specific experimental condition. Simultaneously, among these six strains, three contained a *nahE* gene and one carried a *nahAc* gene, demonstrating that a single biomarker, either *nahAc* or *nahE*, could not be an entirely reliable indicator for PAH degraders. Moreover, the two predicted PAH degraders indeed had an incomplete *nah* or *nar* gene cluster, hinting that the poor performance was probably due to the functional gene deletion. Meanwhile, the result also suggested that random forest analysis might be aggressive to some extent when applied to predict biological traits because every enzyme involved in PAH biodegradation was indispensable.

## Conclusions

In the present study, a comprehensive bacterial PAH-degrading gene database was established, and a genome-centric portrait of bacterial PAH-degrading competency was depicted. Then, a global view of PAH-catabolic genes’ phylogenetic distribution was investigated in the public database, showing a wide distribution in *Proteobacteria* and *Actinobacteria*. Simultaneously, seven potential novel PAH-degrader lineages were observed since a few strains from these genera born a complete PAH-catabolic gene cluster. Furthermore, random forest analysis was employed to predict potential PAH degraders in the complete genome database. In total, 28 strains were predicted as potential new PAH degraders, including nine strains encoding a complete PAH-degrading pathway.

Nevertheless, we have to keep in mind that gene expression involves the coordination of multiple biological traits, such as regulators, promoters, and genes encoding lower pathways. Meanwhile, compared to aerobic bacteria-mediated PAH biodegradation, it has been reported that PAHs can also be biodegraded by anaerobic bacteria [86–88], fungi [89], halophilic archaea [71], and microalgae [90] via significantly different pathways. These factors were not considered in the present study, requiring more experimental evidence and studies to move forward. Likewise, the accuracy of this machine learning-based and function-orientated method needs to be further evaluated by experiments. Nevertheless, we believe the method presented in this study could facilitate the exploration of alternative PAH-degrading genes, enzymes, and novel degradation mechanisms.

## Supplementary Information

**Additional file 1.** Details of 95 identified PAH-degrading bacterial strains.

**Additional file 2.** The summary of protein sequences in the PAH-degrading gene database. Detailed information of 225 strains with complete/near-complete PAH-degrading gene cluster.

**Additional file 3.** Supplementary method and figures.

## Data Availability

The datasets analyzed during the current study are downloaded from the NCBI database (updated in March 2021). All in-house scripts used in this study are available at https://github.com/HuangYue-Emma/PAH-biodegradation.

## Abbreviations

GA: Gathering threshold
HMM: Profile hidden markov model
KEGG: Kyoto encyclopedia of genes and genomes
NCBI: National center biotechnology information
ORF: Open reading frame
PAH: Polycyclic aromatic hydrocarbon
RHDs: Ring-hydroxylating dioxygenases
