# Supplementary material for: Polycyclic aromatic hydrocarbon (PAH) biodegradation capacity revealed by a genome-function relationship approach
Source: Environ Microbiome. 2023 Apr 30;18:39. doi: 10.1186/s40793-023-00497-7 (PMC10150532; doi:10.1186/s40793-023-00497-7)
Supplement: Supplementary file 1 — Additional file 1. Details of 95 identified PAH-degrading bacterial strains. [file 40793_2023_497_MOESM1_ESM.docx]

**Table S1. PAH-degrading strains reported in previous studies.**

| **#** | **Stain name** | **Type** | **Country** | **Substrate** | **Taxonomy ID** | **Accession number** | **Level** | **Ref.** |
| --- | --- | --- | --- | --- | --- | --- | --- | --- |
| 1 | *Achromobacter denitrificans* strain PheN1 | 5 | Singapore | Phn | 32002 | GCA_003812265.1 | Complete | [1] |
| 2 | *Acidovorax carolinensis* strain NA2 | 1 | US | Phn; Naph; Chr; B[a]A | 553814 | GCA_002157165.1 | Complete | [2] |
| 3 | *Acidovorax carolinensis* strain NA3 | 1 | US | Phn; Naph; Chr; B[a]A | 553814 | GCA_002157145.1 | Complete | [2] |
| 4 | *Acidovorax carolinensis* strain P3 | 1 | US | Phn | 553814 | GCA_002157185.1 | Complete | [3] |
| 5 | *Acidovorax carolinensis* strain P4 | 1 | US | Phn | 553814 | GCA_002157125.1 | Complete | [3] |
| 6 | *Alteromonas naphthalenivorans* strain SN2 | 3 | Korea | Phn | 715451 | GCA_000213655.1 | Complete | [4] |
| 7 | *Amycolatopsis methanolica* 239 | 1 | Papua New Guinea | Naph, Xyl | 1068978 | GCA_000739085.1 | Complete | [5] |
| 8 | *Burkholderia multivorans* strain DDS 15A-1 | 5 | Australia |  | 87883 | GCA_000756005.1 | Complete | [6] |
| 9 | *Celeribacter indicus* strain P73 | 3 | Indian Ocean | Naph; Phn; Ant; Fla | 1208324 | GCA_000819565.1 | Complete | [7] |
| 10 | *Croceicoccus naphthovorans* strain PQ-2 | 3 | China | Naph | 1348774 | GCA_001028705.1 | Complete | [8] |
| 11 | *Cycloclasticus* sp. P1 | 3 | west Pacific | Pyr | 385025 | GCA_000299965.1 | Complete | [9] |
| 12 | *Cycloclasticus* sp. PY97N | 3 | China Yellow sea | Pyr; Fla | 728003 | GCA_002443175.1 | Complete | [10] |
| 13 | *Cycloclasticus zancles* 78-ME | 3 | Italy | Pyr | 1198232 | GCA_000442595.1 | Complete | [11] |
| 14 | *Delftia* sp. Cs1-4 | 1 | US | Phn | 742013 | GCA_000214395.1 | Complete | [12] |
| 15 | *Erythrobacter atlanticus* strain s21-N3 | 3 | Atlantic Ocean | Naph; Phn; Pyr | 1648404 | GCA_001077815.2 | Complete | [13] |
| 16 | *Immundisolibacter cernigliae* strain TR3.2 | 1 | US | Pyr | 1810504 | GCA_001697225.1 | Complete | [14] |
| 17 | *Martelella* sp. AD-3 | 1 | China | Ant; Phn | 686597 | GCA_001578105.1 | Complete | [15] |
| 18 | *Massilia* sp. WG5 | 1 | China | Phn | 1707785 | GCA_001412595.2 | Complete | [16] |
| 19 | *Mycobacterium* sp. EPa45 | 1 | Japan | Phn | 1545728 | GCA_001021385.1 | Complete | [17] |
| 20 | *Mycobacterium* sp. KMS* | 1 | US | Pyr; Phn | 189918 | GCA_000015405.1 | Complete | [18] |
| 21 | *Mycobacterium* sp. PYR15 | 3 | Korea | Pyr | 2051552 | GCA_002335685.1 | Complete | [19] |
| 22 | *Mycobacterium* sp. WY10 | 1 | China | Phn; Pyr | 1920667 | GCA_001886515.1 | Complete | [20] |
| 23 | *Mycolicibacterium gilvum* PYR-GCK* | 2 | US | Pyr; Phn | 350054 | GCA_000016365.1 | Complete | [21] |
| 24 | *Mycolicibacterium gilvum* Spyr1 | 1 | Greece | Pyr; Phn | 278137 | GCA_000184435.1 | Complete | [22] |
| 25 | *Mycolicibacterium rhodesiae* NBB3 | 3 | Australia |  | 710685 | GCA_000230895.3 | Complete | [23] |
| 26 | *Mycolicibacterium vanbaalenii* PYR-1 | 3 | US | Pyr; Phn; Fla; Naph; Ant | 350058 | GCA_000015305.1 | Complete | [24] |
| 27 | *Novosphingobium aromaticivorans* DSM 12444 | 3 | Atlantic Ocean | Naph; Biph | 279238 | GCA_000013325.1 | Complete | {Balkwill, 1997 #107}[25, 26] |
| 28 | *Novosphingobium pentaromativorans* US6-1 | 3 | Korea | Naph; Phn; Ant; Pyr; B[a]p | 1088721 | GCA_000767465.1 | Complete | [27] |
| 29 | *Novosphingobium* sp. PP1Y | 3 | Italy | Naph; Phn; Ant; Pyr | 702113 | GCA_000253255.1 | Complete | [28] |
| 30 | *Paraburkholderia aromaticivorans* strain BN5 | 1 | Korea | Naph | 2026199 | GCA_002278075.1 | Complete | [29] |
| 31 | *Polaromonas naphthalenivorans* CJ2 | 2 | US | Naph | 365044 | GCA_000015505.1 | Complete | [30] |
| 32 | *Polymorphum gilvum* SL003B-26A1 | 1 | China | Biph; crude oil | 991905 | GCA_000192745.1 | Complete | [31] |
| 33 | *Pseudarthrobacter phenanthrenivorans* Sphe3 | 1 | Greece | Phn; Ant | 930171 | GCA_000189535.1 | Complete | [32] |
| 34 | *Pseudomonas balearica* DSM 6083 | 3 | Spain | Naph | 1123016 | GCA_000818015.1 | Complete | [33] |
| 35 | *Pseudomonas citronellolis strain* SJTE-3 | 4 | China | Naph; Phn; Fla; Ant | 53408 | GCA_001654435.1 | Complete | [34] |
| 36 | *Pseudomonas frederiksbergensis strain* AS1 | 1 | Korea | Naph | 104087 | GCA_001952935.1 | Complete | [35] |
| 37 | *Pseudomonas putida* ND6 | 4 | China | Naph; Phn | 231023 | GCA_000264665.2 | Complete | [36] |
| 38 | *Pseudomonas putida* strain BS3701 | 1 | Russia | Naph | 303 | GCA_013425825.1 | Complete | [37] |
| 39 | *Pseudomonas* sp. MPDS | 1 | China | Naph; Flu | 2762896 | GCA_013283895.1 | Complete | [38] |
| 40 | *Pseudomonas stutzeri* CCUG 29243 | 3 | Spain | Naph | 1196835 | GCA_000267545.1 | Complete | [39] |
| 41 | *Pseudomonas stutzeri* strain 19SMN4 | 3 | Spain |  | 316 | GCA_000661915.1 | Complete | [40] |
| 42 | *Rhodococcus opacus* B4 | 1 | Japan | Naph | 632772 | GCA_000010805.1 | Complete | [41] |
| 43 | *Rhodococcus* sp. WAY2 | 1 | Spain | Naph; Xyl; Biph | 2663121 | GCA_009859915.1 | Complete | [42] |
| 44 | *Rhodococcus* sp. WB9 | 1 | China | Phn | 2594007 | GCA_007558985.1 | Complete | [43] |
| 45 | *Rugosibacter aromaticivorans* strain Ca6 | 1 | US | Phn; Pyr | 1565605 | GCA_000934545.1 | Complete | [44] |
| 46 | *Sphingobium barthaii* strain KK22 | 1 | US | B[a]A; Naph; Phn | 336203 | GCA_015074805.1 | Complete | [45] |
| 47 | *Sphingobium yanoikuyae* strain SJTF8 | 4 | China | Naph; Phn; Fla; Ant | 13690 | GCA_003697265.1 | Complete | [34] |
| 48 | *Mycobacterium* sp. JLS* | 1 | US | Pyr; Phn | / | GCA_000016005.1 | Complete | [18] |
| 49 | *Mycobacterium* sp. MCS* | 1 | US | Pyr; Phn | / | GCA_000014165.1 | Complete | [18] |
| 50 | *Burkholderia* sp. HB1* | 1 | Japan | Phn | / | GCA_001293045.1 | Complete | [46] |
| 51 | *Geobacillus* sp. JF8 | 4 | Japan | Naph; Biph | 1921421 | GCA_000445995.2 | Complete | [47] |
| 52 | *Sphingomonas aromaticivorans* B0695 | 1 | US | Ant; Phn; Fla | 279238 | GCA_000013325.1 | Complete | [48] |
| 53 | *Pseudomonas aeruginosa* PaK1 | 4 | China | Naph | 1009714 | GCA_902172305.1 | Complete | [49] |
| 54 | *Mycobacterium frederiksbergense* FAn9 | 1 | Denmark | Pyr; Phn | 117567 | GCA_012223425.1 | Chromosome | [50] |
| 55 | *Rhodococcus opacus* R7 | 1 | Italy | Naph | 37919 | GCA_000736435.1 | Chromosome | [51] |
| 56 | *Advenella kashmirensis* strain W13003 | 3 | China | Phn; Pyr | 1424334 | GCA_000506985.1 | Scaffold | [52] |
| 57 | *Arthrobacter sp.* SF27 | 1 | Russia | Naph; Phn; Bioph | 1663 | GCA_012952295.1 | Scaffold | [53] |
| 58 | *Burkholderia* sp. C3 | 1 | US, Hawai | Phn; Naph | 28450 | GCA_001197775.1 | Scaffold | [54] |
| 59 | *Burkholderia* sp. Ch1-1 |  | US | Phn | 243261 | GCA_000178415.2 | Scaffold | [55] |
| 60 | *Cycloclasticus* sp. PY97M | 3 | China Yellow sea | Pyr; Fla | 34068 | GCA_000444935.1 | Scaffold | [10] |
| 61 | *Croceicoccus* *pelagius* Ery9 | 3 | Atlantic Ocean | Phn | 170334 | GCA_001661915.1 | Scaffold | [56] |
| 62 | *Marinobacter nanhaiticus* D15-8W | 3 | South China Sea | Naph; Phn; Ant | 626887 | GCA_000364845.1 | Scaffold | [57] |
| 63 | *Novosphingobium* sp. HR1a |  |  | Naph; phn; Pry | 1395637 | GCA_015594425.1 | Scaffold | [58] |
| 64 | *Pseudomonas stutzeri* KOS6 | 4 | Russia | Naph; Phn | 1218352 | GCA_000307775.2 | Scaffold | [59] |
| 65 | *Rhodococcus ruber* Chol-4 | 4 | Spain | Naph | 1240349 | GCA_000347955.2 | Scaffold | [60] |
| 66 | *Sphingobium yanoikuyae* B1 | 1 | US | Naph; Phn; Ant; Bpn | 13690 | GCA_000735675.1 | Scaffold | [61, 62] |
| 67 | *Sphingomonas* sp. strain Ant20 | 1 | Antarctica | Ant | 104605 | GCA_000783375.1 | Scaffold | [63] |
| 68 | *Algiphilus aromaticivorans* DG1253 | 3 | US | Naph; Phn; Ant | 1415780 | GCA_000733765.1 | Contig | [64] |
| 69 | *Arenibacter algicola* sp. TG409 | 3 | US | Naph; Phn | 616991 | GCA_000733925.1 | Contig | [65] |
| 70 | *Diaphorobacter* sp. DS2 | 4 | India | 3NT; Naph | 1302548 | GCA_004563745.1 | Contig | [66] |
| 71 | *delta proteobacterium* NaphS2 | 3 | Germany | Naph | 88274 | GCA_000179315. | Contig | [67] |
| 72 | *Herbaspirillum* sp. RV1423 | 1 | German | Naph | 1443993 | GCA_000577615.1 | Contig | [68] |
| 73 | *Hydrocarboniphaga effusa* AP103 | 1 | US |  | 1172194 | GCA_000271305.1 | Contig | [69] |
| 74 | *Marinomonas* sp. D104 | 3 | Arctic Ocean | Naph; Phn; Ant; Pyr | 1208321 | GCA_000508165.1 | Contig | [70] |
| 75 | *Mycobacterium aromaticivorans* JS19b1 | 1 | US-Hawaii | Phn(Fla-; Pyr-) | 1440774 | GCA_000559085.2 | Contig | [71] |
| 76 | *Mycobacterium* sp. PYR10* | 3 | Korea | Pyr | 2051551 | GCA_002762075.1 | Contig | [19] |
| 77 | *Massilia sp.* WF1 | 1 | China | Phn | 1406431 | GCA_001028775.2 | Contig | [72] |
| 78 | *Oceanicola* sp. MCTG156(1a)* | 3 | UK | Phn | 1415756 | GCA_000744955.1 | Contig | [73] |
| 79 | *Nocardia farcinica* TRH1* | 3 | Brazil | Phn; Pyr; Ant | 37329 | GCA_001704135.1 | Contig | [74] |
| 80 | *Polycyclovorans algicola* TG408 | 3 | US | Naph; Phn; Ant | 1415779 | GCA_000711245.1 | Contig | [75] |
| 81 | *Porticoccus hydrocarbonoclasticus* strain MCTG13d | 3 | US | Phn; Ant; Pyr | 1415778 | GCA_000744735.1 | Contig | [76] |
| 82 | *Pseudomonas putida* CSV86 | 1 | India | Naph | 1005395 | GCA_000319305.2 | Contig | [77, 78] |
| 83 | *Pseudomonas putida* OUS82 | 1 | Japan | Phn; Naph | 1410669 | GCA_000507325.1 | Contig | [79] |
| 84 | *Pseudomonas aeruginosa* DQ8 | 1 | China | Phn; Pyr | 1211113 | GCA_000283055.1 | Contig | [80] |
| 85 | *Rhizobium* sp. TSY03b | 2 | Japan | Naph | 1118451 | GCA_900492205.1 | Contig | [81] |
| 86 | *Rhodococcus imtechensis* RKJ300 | 1 | India | Naph | 1165867 | GCA_000260815.1 | Contig | [82] |
| 87 | *Rhodococcus opacus* M213 | 1 | US | Naph | 1129896 | GCA_000264745.2 | Contig | [83] |
| 88 | *Rhodococcus* sp. NCIMB12038 | 1 | UK | Naph | 933800 | GCA_002165735.1 | Contig | [84, 85] |
| 89 | *Rhodococcus wratislaviensis* IFP 2016 | 5 | France | Naph | 1195242 | GCA_000325625.1 | Contig | [86] |
| 90 | *Rhodovulum* sp. NI22* | 3 | US | Naph | 1469613 | GCA_000756565.1 | Contig | [87] |
| 91 | *Sphingobium* sp. Ant17 | 1 | Antaretica | Phn | 1461752 | GCA_000588875.1 | Contig | [88] |
| 92 | *Sphingobium* sp. C100 | 3 | ARctic Ocean | Naph; Ant; Fla | 1207055 | GCA_000508185.1 | Contig | [89] |
| 93 | *Sphingomonas paucimobilis* 20006FA | 1 | Argentina | Phn | 2056888 | GCA_001651765.1 | Contig | [90] |
| 94 | *Sphingomonas* sp. LH128* | 1 | Belgium | Pyr; Chr; B[a]A; Phn | 473781 | GCA_000293195.2 | Contig | [91] |
| 95 | *Thioclava dalianensis* DLFJ1-1 | 2 | China | Oil | 1185766 | GCA_000715505.1 | Contig | [92] |

Strain name: ***** Anomalous assembly.

Type: 1. Soil and underground water; 2. River and sediments; 3. Sea and sediments; 4. Wastewater treatment plans; 5. Others.

Substrate: Naphthalene (Naph); Phenanthrene (Phn); Anthracene (Ant); Fluorene (Flu); Fluoranthene (Fla); Pyrene (Pyr); Xylene (Xyl); Benz[a]anthracene (B[a]A); Chrysene (Chr); Biphenyls (Biph); 3-nitrotoluene (3NT).

**Reference:**

1. Zhang Z, Sun J, Guo H, Wang C, Fang T, Rogers MJ, et al. Anaerobic biodegradation of phenanthrene by a newly isolated nitrate-dependent *Achromobacter denitrificans* strain PheN1 and exploration of the biotransformation processes by metabolite and genome analyses. Environ Microbiol. 2021;23:908-923.

2. Singleton DR, Ramirez LG and Aitken MD. Characterization of a polycyclic aromatic hydrocarbon degradation gene cluster in a phenanthrene-degrading *Acidovorax* strain. Appl Environ Microbiol. 2009;75:2613-2620.

3. Singleton DR, Lee J, Dickey AN, Stroud A, Scholl EH, Wright FA, et al. Polyphasic characterization of four soil-derived phenanthrene-degrading *Acidovorax* strains and proposal of *Acidovorax carolinensis* sp. nov. Syst Appl Microbiol. 2018;41:460-472.

4. Jin HM, Jeong H, Moon EJ, Math RK, Lee K, Kim HJ, et al. Complete genome sequence of the polycyclic aromatic hydrocarbon-degrading bacterium *Alteromonas* sp. strain SN2. J Bacteriol. 2011;193:4292-4293.

5. Bourguignon N, Bargiela R, Rojo D, Chernikova TN, de Rodas SAL, García-Cantalejo J, et al. Insights into the degradation capacities of *Amycolatopsis tucumanensis* DSM 45259 guided by microarray data. World J Microbiol Biotechnol. 2016;32:1-12.

6. Daligault H, Davenport K, Minogue T, Bishop-Lilly K, Broomall S, Bruce D, et al. Whole-genome assemblies of 56 *Burkholderia* species. Genome announcements. 2014;2:e01106-01114.

7. Lai Q, Cao J, Yuan J, Li F and Shao Z. *Celeribacter indicus* sp. nov., a polycyclic aromatic hydrocarbon-degrading bacterium from deep-sea sediment and reclassification of *Huaishuia halophila* as *Celeribacter halophilus* comb. nov. Int J Syst Evol Microbiol. 2014;64:4160-4167.

8. Huang Y, Zeng Y, Feng H, Wu Y and Xu X. *Croceicoccus naphthovorans* sp. nov., a polycyclic aromatic hydrocarbons-degrading and acylhomoserine-lactone-producing bacterium isolated from marine biofilm, and emended description of the genus *Croceicoccus*. Int J Syst Evol Microbiol. 2015;65:1531-1536.

9. Wang B, Lai Q, Cui Z, Tan T and Shao Z. A pyrene-degrading consortium from deep-sea sediment of the West Pacific and its key member *Cycloclasticus* sp. P1. Environ Microbiol. 2008;10:1948-1963.

10. Cui Z, Xu G, Li Q, Gao W and Zheng L. Genome Sequence of the Pyrene- and Fluoranthene-Degrading Bacterium *Cycloclasticus* sp. Strain PY97M. Genome Announc. 2013;1.

11. Messina E, Denaro R, Crisafi F, Smedile F, Cappello S, Genovese M, et al. Genome sequence of obligate marine polycyclic aromatic hydrocarbons-degrading bacterium *Cycloclasticus* sp. 78-ME, isolated from petroleum deposits of the sunken tanker Amoco Milford Haven, Mediterranean Sea. Mar Genomics. 2016;25:11-13.

12. Chen S and Hickey WJ. Development of Tools for Genetic Analysis of Phenanthrene Degradation and Nanopod Production by *Delftia* sp. Cs1-4. Front microbiol. 2011;2:187.

13. Zhuang L, Liu Y, Wang L, Wang W and Shao Z. *Erythrobacter atlanticus* sp. nov., a bacterium from ocean sediment able to degrade polycyclic aromatic hydrocarbons. Int J Syst Evol Microbiol. 2015;65:3714-3719.

14. Corteselli EM, Aitken MD and Singleton DR. Description of *Immundisolibacter cernigliae* gen. nov., sp. nov., a high-molecular-weight polycyclic aromatic hydrocarbon-degrading bacterium within the class *Gammaproteobacteria*, and proposal of *Immundisolibacterales* ord. nov. and *Immundisolibacteraceae* fam. nov. Int J Syst Evol Microbiol. 2017;67:925-931.

15. Cui C-Z, Feng T-C, Yu Y-Q, Dong F, Yang X-M, Feng Y-Y, et al. Isolation, charcaterization of an anthracene degrading bacterium *Martelella* sp. AD-3 and cloning of dioxygenase gene. Huan jing ke xue= Huanjing kexue. 2012;33:4062-4068.

16. Lou J, Gu H, Wang H, An Q and Xu J. Complete genome sequence of *Massilia* sp. WG5, an efficient phenanthrene-degrading bacterium from soil. J Biotechnol. 2016;218:49-50.

17. Kato H, Ogawa N, Ohtsubo Y, Oshima K, Toyoda A, Mori H, et al. Complete Genome Sequence of a Phenanthrene Degrader, *Mycobacterium* sp. Strain EPa45 (NBRC 110737), Isolated from a Phenanthrene-Degrading Consortium. Genome Announc. 2015;3.

18. Miller CD, Hall K, Liang YN, Nieman K, Sorensen D, Issa B, et al. Isolation and characterization of polycyclic aromatic hydrocarbon-degrading *Mycobacterium* isolates from soil. Microb Ecol. 2004;48:230-238.

19. Kim DW, Lee K, Lee DH and Cha CJ. Comparative genomic analysis of pyrene-degrading *Mycobacterium* species: Genomic islands and ring-hydroxylating dioxygenases involved in pyrene degradation. J Microbiol. 2018;56:798-804.

20. Sun S, Wang H, Chen Y, Lou J, Wu L and Xu J. Salicylate and phthalate pathways contributed differently on phenanthrene and pyrene degradations in *Mycobacterium* sp. WY10. J Hazard Mater. 2019;364:509-518.

21. Dean-Ross D and Cerniglia C. Degradation of pyrene by Mycobacterium flavescens. Applied Microbiology and Biotechnology. 1996;46:307-312.

22. Karabika E, Kallimanis A, Dados A, Pilidis G, Drainas C and Koukkou AI. Taxonomic identification and use of free and entrapped cells of a new *Mycobacterium* sp., strain Spyr1 for degradation of polycyclic aromatic hydrocarbons (PAHs). Appl Biochem Biotechnol. 2009;159:155-167.

23. Coleman NV, Bui NB and Holmes AJ. Soluble di-iron monooxygenase gene diversity in soils, sediments and ethene enrichments. Environ Microbiol. 2006;8:1228-1239.

24. Heitkamp MA, Franklin W and Cerniglia CE. Microbial metabolism of polycyclic aromatic hydrocarbons: isolation and characterization of a pyrene-degrading bacterium. Appl Environ Microbiol. 1988;54:2549-2555.

25. Balkwill DL, Drake GR, Reeves RH, Fredrickson JK, White DC, Ringelberg DB, et al. Taxonomic study of aromatic-degrading bacteria from deep-terrestrial-subsurface sediments and description of *Sphingomonas aromaticivorans* sp. nov., *Sphingomonas subterranea* sp. nov., and *Sphingomonas stygia* sp. nov. Int J Syst Bacteriol. 1997;47:191-201.

26. Romine MF, Stillwell LC, Wong K-K, Thurston SJ, Sisk EC, Sensen C, et al. Complete sequence of a 184-kilobase catabolic plasmid from *Sphingomonas aromaticivorans* F199. J Bacteriol. 1999;181:1585-1602.

27. Sohn JH, Kwon KK, Kang JH, Jung HB and Kim SJ. *Novosphingobium pentaromativorans* sp. nov., a high-molecular-mass polycyclic aromatic hydrocarbon-degrading bacterium isolated from estuarine sediment. Int J Syst Evol Microbiol. 2004;54:1483-1487.

28. Notomista E, Pennacchio F, Cafaro V, Smaldone G, Izzo V, Troncone L, et al. The marine isolate *Novosphingobium* sp. PP1Y shows specific adaptation to use the aromatic fraction of fuels as the sole carbon and energy source. Microb Ecol. 2011;61:582-594.

29. Lee Y, Lee Y and Jeon CO. Biodegradation of naphthalene, BTEX, and aliphatic hydrocarbons by *Paraburkholderia aromaticivorans* BN5 isolated from petroleum-contaminated soil. Sci Rep. 2019;9:860.

30. Jeon CO, Park M, Ro HS, Park W and Madsen EL. The naphthalene catabolic (*nag*) genes of *Polaromonas naphthalenivorans* CJ2: evolutionary implications for two gene clusters and novel regulatory control. Appl Environ Microbiol. 2006;72:1086-1095.

31. Li SG, Tang YQ, Nie Y, Cai M and Wu XL. Complete genome sequence of *Polymorphum gilvum* SL003B-26A1T, a crude oil-degrading bacterium from oil-polluted saline soil. J Bacteriol. 2011;193:2894-2895.

32. Kallimanis A, Kavakiotis K, Perisynakis A, Sproer C, Pukall R, Drainas C, et al. *Arthrobacter phenanthrenivorans* sp. nov., to accommodate the phenanthrene-degrading bacterium *Arthrobacter* sp. strain Sphe3. Int J Syst Evol Microbiol. 2009;59:275-279.

33. Bennasar-Figueras A, Salva-Serra F, Jaen-Luchoro D, Segui C, Aliaga F, Busquets A, et al. Complete Genome Sequence of *Pseudomonas* balearica DSM 6083T. Genome Announc. 2016;4.

34. Zheng D, Wang X, Wang P, Peng W, Ji N and Liang R. Genome Sequence of *Pseudomonas citronellolis* SJTE-3, an Estrogen- and Polycyclic Aromatic Hydrocarbon-Degrading Bacterium. Genome Announc. 2016;4.

35. Kim J and Park W. Genome Analysis of Naphthalene-Degrading *Pseudomonas* sp. AS1 Harboring the Megaplasmid pAS1. J Microbiol Biotechnol. 2018;28:330-337.

36. Zhao H, Chen D, Li Y and Cai B. Overexpression, purification and characterization of a new salicylate hydroxylase from naphthalene-degrading *Pseudomonas* sp. strain ND6. Microbiol Res. 2005;160:307-313.

37. Pozdnyakova-Filatova I, Petrikov K, Vetrova A, Frolova A, Streletskii R and Zakharova M. The naphthalene catabolic genes of *Pseudomonas putida* BS3701: Additional regulatory control. Front microbiol. 2020;11:1217.

38. Liu Y, Hu H, Zanaroli G, Xu P and Tang H. A *Pseudomonas* sp. strain uniquely degrades PAHs and heterocyclic derivatives via lateral dioxygenation pathways. J Hazard Mater. 2021;403:123956.

39. Brunet-Galmes I, Busquets A, Pena A, Gomila M, Nogales B, Garcia-Valdes E, et al. Complete genome sequence of the naphthalene-degrading bacterium *Pseudomonas stutzeri* AN10 (CCUG 29243). J Bacteriol. 2012;194:6642-6643.

40. Rosselló R, Garcia-Valdes E, Lalucat J and Ursing J. Genotypic and phenotypic diversity of *Pseudomonas stutzeri*. Syst Appl Microbiol. 1991;14:150-157.

41. Na KS, Kuroda A, Takiguchi N, Ikeda T, Ohtake H and Kato J. Isolation and characterization of benzene-tolerant *Rhodococcus opacus* strains. J Biosci Bioeng. 2005;99:378-382.

42. Garrido-Sanz D, Sansegundo-Lobato P, Redondo-Nieto M, Suman J, Cajthaml T, Blanco-Romero E, et al. Analysis of the biodegradative and adaptive potential of the novel polychlorinated biphenyl degrader *Rhodococcus* sp. WAY2 revealed by its complete genome sequence. Microb Genom. 2020;6.

43. Sun S, Wang H, Fu B, Zhang H, Lou J, Wu L, et al. Non-bioavailability of extracellular 1-hydroxy-2-naphthoic acid restricts the mineralization of phenanthrene by *Rhodococcus* sp. WB9. Sci Total Environ. 2020;704:135331.

44. Corteselli EM, Aitken MD and Singleton DR. Rugosibacter aromaticivorans gen. nov., sp. nov., a bacterium within the family Rhodocyclaceae, isolated from contaminated soil, capable of degrading aromatic compounds. International journal of systematic and evolutionary microbiology. 2017;67:311.

45. Kunihiro M, Ozeki Y, Nogi Y, Hamamura N and Kanaly RA. Benz[*a*]anthracene biotransformation and production of ring fission products by *Sphingobium* sp. strain KK22. Appl Environ Microbiol. 2013;79:4410-4420.

46. Ohtsubo Y, Moriya A, Kato H, Ogawa N, Nagata Y and Tsuda M. Complete Genome Sequence of a Phenanthrene Degrader, *Burkholderia* sp. HB-1 (NBRC 110738). Genome Announc. 2015;3.

47. Miyazawa D, Thanh LTH, Tani A, Shintani M, Loc NH, Hatta T, et al. Isolation and Characterization of Genes Responsible for Naphthalene Degradation from Thermophilic Naphthalene Degrader, Geobacillus sp. JF8. Microorganisms. 2020;8:44.

48. Shi T, Fredrickson JK and Balkwill DL. Biodegradation of polycyclic aromatic hydrocarbons by *Sphingomonas* strains isolated from the terrestrial subsurface. J Ind Microbiol Biotechnol. 2001;26:283-289.

49. Takizawa N, Iida T, Sawada T, Yamauchi K, Wang Y-W, Fukuda M, et al. Nucleotide sequences and characterization of genes encoding naphthalene upper pathway of *Pseudomonas aeruginosa* PaK1 and *Pseudomonas putida* OUS82. J Biosci Bioeng. 1999;87:721-731.

50. Willumsen P, Karlson U, Stackebrandt E and Kroppenstedt RM. *Mycobacterium frederiksbergense* sp. nov., a novel polycyclic aromatic hydrocarbon-degrading *Mycobacterium* species. Int J Syst Evol Microbiol. 2001;51:1715-1722.

51. Di Gennaro P, Rescalli E, Galli E, Sello G and Bestetti G. Characterization of *Rhodococcus opacus* R7, a strain able to degrade naphthalene and o-xylene isolated from a polycyclic aromatic hydrocarbon-contaminated soil. Res Microbiol. 2001;152:641-651.

52. Wang X, Jin D, Zhou L, Wu L, An W and Zhao L. Draft Genome Sequence of *Advenella kashmirensis* Strain W13003, a Polycyclic Aromatic Hydrocarbon-Degrading Bacterium. Genome Announc. 2014;2.

53. Plotnikova E, Altyntseva O, Kosheleva I, Puntus I, Filonov A, Gavrish EY, et al. Bacterial degraders of polycyclic aromatic hydrocarbons isolated from salt-contaminated soils and bottom sediments in salt mining areas. Microbiology. 2001;70:51-58.

54. Seo JS, Keum YS, Hu Y, Lee SE and Li QX. Degradation of phenanthrene by *Burkholderia* sp. C3: initial 1,2- and 3,4-dioxygenation and *meta*- and *ortho*-cleavage of naphthalene-1,2-diol. Biodegradation. 2007;18:123-131.

55. Vacca D, Bleam W and Hickey W. Isolation of soil bacteria adapted to degrade humic acid-sorbed phenanthrene. Appl Environ Microbiol. 2005;71:3797-3805.

56. Wu Y-H, Li G-Y, Jian S-L, Cheng H, Huo Y-Y, Wang C-S, et al. *Croceicoccus pelagius* sp. nov. and Croceicoccus mobilis sp. nov., isolated from marine environments. Int J Syst Evol Microbiol. 2016;66:4506-4511.

57. Cui Z, Gao W, Li Q, Xu G and Zheng L. Genome Sequence of the Polycyclic Aromatic Hydrocarbon-Degrading Bacterium Strain *Marinobacter nanhaiticus* D15-8W^T^. Genome Announc. 2013;1.

58. Segura A, Hernandez-Sanchez V, Marques S and Molina L. Insights in the regulation of the degradation of PAHs in *Novosphingobium* sp. HR1a and utilization of this regulatory system as a tool for the detection of PAHs. Sci Total Environ. 2017;590-591:381-393.

59. Grigoryeva TV, Laikov AV, Naumova RP, Manolov AI, Larin AK, Karpova IY, et al. Draft Genome of the Nitrogen-Fixing Bacterium *Pseudomonas stutzeri* Strain KOS6 Isolated from Industrial Hydrocarbon Sludge. Genome Announc. 2013;1.

60. Fernandez de Las Heras L, Garcia Fernandez E, Maria Navarro Llorens J, Perera J and Drzyzga O. Morphological, physiological, and molecular characterization of a newly isolated steroid-degrading actinomycete, identified as *Rhodococcus ruber* strain Chol-4. Curr Microbiol. 2009;59:548-553.

61. Pinyakong O, Habe H and Omori T. The unique aromatic catabolic genes in sphingomonads degrading polycyclic aromatic hydrocarbons (PAHs). J Gen Appl Microbiol. 2003;49:1-19.

62. Khan AA, Wang R-F, Cao W-W, Franklin W and Cerniglia CE. Reclassification of a polycyclic aromatic hydrocarbon-metabolizing bacterium, *Beijerinckia* sp. strain B1, as *Sphingomonas yanoikuyae* by fatty acid analysis, protein pattern analysis, DNA-DNA hybridization, and 16S ribosomal DNA sequencing. Int J Syst Evol Microbiol. 1996;46:466-469.

63. Ronca S, Frossard A, Guerrero LD, Makhalanyane TP, Aislabie JM and Cowan DA. Draft genome sequence of *Sphingomonas* sp. strain Ant20, isolated from oil-contaminated soil on Ross Island, Antarctica. Genome Announcements. 2015;3:e01309-01314.

64. Gutierrez T, Green DH, Whitman WB, Nichols PD, Semple KT and Aitken MD. *Algiphilus aromaticivorans* gen. nov., sp. nov., an aromatic hydrocarbon-degrading bacterium isolated from a culture of the marine dinoflagellate Lingulodinium polyedrum, and proposal of *Algiphilaceae* fam. nov. Int J Syst Evol Microbiol. 2012;62:2743-2749.

65. Gutierrez T, Rhodes G, Mishamandani S, Berry D, Whitman WB, Nichols PD, et al. Polycyclic aromatic hydrocarbon degradation of phytoplankton-associated *Arenibacter* spp. and description of *Arenibacter algicola* sp. nov., an aromatic hydrocarbon-degrading bacterium. Appl Environ Microbiol. 2014;80:618-628.

66. Methods N, Singh D, Kumari A and Ramanathan G. 3-Nitrotoluene dioxygenase from *Diaphorobacter* sp. strains: cloning, sequencing and evolutionary studies. Biodegradation. 2014;25:479-492.

67. Galushko A, Minz D, Schink B and Widdel F. Anaerobic degradation of naphthalene by a pure culture of a novel type of marine sulphate-reducing bacterium. Environ Microbiol. 1999;1:415-420.

68. Jauregui R, Rodelas B, Geffers R, Boon N, Pieper DH and Vilchez-Vargas R. Draft Genome Sequence of the Naphthalene Degrader *Herbaspirillum* sp. Strain RV1423. Genome Announc. 2014;2.

69. Palleroni NJ, Port AM, Chang H-K and Zylstra GJ. *Hydrocarboniphaga effusa* gen. nov., sp. nov., a novel member of the *γ*-Proteobacteria active in alkane and aromatic hydrocarbon degradation. Int J Syst Evol Microbiol. 2004;54:1203-1207.

70. Dong C, Bai X, Lai Q, Xie Y, Chen X and Shao Z. Draft Genome Sequence of *Marinomonas* sp. Strain D104, a Polycyclic Aromatic Hydrocarbon-Degrading Bacterium from the Deep-Sea Sediment of the Arctic Ocean. Genome Announc. 2014;2.

71. Hennessee CT, Seo JS, Alvarez AM and Li QX. Polycyclic aromatic hydrocarbon-degrading species isolated from Hawaiian soils: *Mycobacterium crocinum* sp. nov., *Mycobacterium pallens* sp. nov., *Mycobacterium rutilum* sp. nov., *Mycobacterium rufum* sp. nov. and *Mycobacterium aromaticivorans* sp. nov. Int J Syst Evol Microbiol. 2009;59:378-387.

72. Wang H, Lou J, Gu H, Luo X, Yang L, Wu L, et al. Efficient biodegradation of phenanthrene by a novel strain *Massilia* sp. WF1 isolated from a PAH-contaminated soil. Environmental Science and Pollution Research. 2016;23:13378-13388.

73. Gutierrez T, Whitman WB, Huntemann M, Copeland A, Chen A, Vargese N, et al. Genome sequence of *Oceanicola* sp. strain MCTG156 (1a), isolated from a Scottish coastal phytoplankton net sample. Genome announcements. 2017;5:e00796-00717.

74. Rodrigues EM, Vidigal PMP, Pylro VS, Morais DK, Leite LR, Roesch LF, et al. Draft genome of *Nocardia farcinica* TRH1, a linear and polycyclic aromatic hydrocarbon-degrading bacterium isolated from the coast of Trindade Island, Brazil. Braz J Microbiol. 2017;48:391-392.

75. Gutierrez T, Green DH, Nichols PD, Whitman WB, Semple KT and Aitken MD. *Polycyclovorans algicola* gen. nov., sp. nov., an aromatic-hydrocarbon-degrading marine bacterium found associated with laboratory cultures of marine phytoplankton. Appl Environ Microbiol. 2013;79:205-214.

76. Gutierrez T, Nichols PD, Whitman WB and Aitken MD. *Porticoccus hydrocarbonoclasticus* sp. nov., an aromatic hydrocarbon-degrading bacterium identified in laboratory cultures of marine phytoplankton. Appl Environ Microbiol. 2012;78:628-637.

77. Basu A, Apte SK and Phale PS. Preferential utilization of aromatic compounds over glucose by *Pseudomonas putida* CSV86. Appl Environ Microbiol. 2006;72:2226-2230.

78. Mahajan MC, Phale PS and Vaidyanathan CS. Evidence for the involvement of multiple pathways in the biodegradation of 1-and 2-methylnaphthalene by *Pseudomonas putida* CSV86. Arch Microbiol. 1994;161:425-433.

79. Kiyohara H, Torigoe S, Kaida N, Asaki T, Iida T, Hayashi H, et al. Cloning and characterization of a chromosomal gene cluster, pah, that encodes the upper pathway for phenanthrene and naphthalene utilization by *Pseudomonas putida* OUS82. J Bacteriol. 1994;176:2439-2443.

80. Zhang Z, Hou Z, Yang C, Ma C, Tao F and Xu P. Degradation of n-alkanes and polycyclic aromatic hydrocarbons in petroleum by a newly isolated *Pseudomonas aeruginosa* DQ8. Bioresour Technol. 2011;102:4111-4116.

81. Kaiya S, Rubaba O, Yoshida N, Yamada T and Hiraishi A. Characterization of *Rhizobium naphthalenivorans* sp. nov. with special emphasis on aromatic compound degradation and multilocus sequence analysis of housekeeping genes. J Gen Appl Microbiol. 2012;58:211-224.

82. Ghosh A, Khurana M, Chauhan A, Takeo M, Chakraborti AK and Jain RK. Degradation of 4-nitrophenol, 2-chloro-4-nitrophenol, and 2, 4-dinitrophenol by *Rhodococcus imtechensis* strain RKJ300. Environ Sci Technol. 2010;44:1069-1077.

83. Uz I, Duan Y and Ogram A. Characterization of the naphthalene-degrading bacterium, *Rhodococcus opacus* M213. FEMS Microbiol Lett. 2000;185:231-238.

84. Kulakov LA, Allen CC, Lipscomb DA and Larkin MJ. Cloning and characterization of a novel *cis*-naphthalene dihydrodiol dehydrogenase gene (*narB*) from *Rhodococcus* sp. NCIMB12038. FEMS Microbiol Lett. 2000;182:327-331.

85. Larkin MJ, Allen CC, Kulakov LA and Lipscomb DA. Purification and characterization of a novel naphthalene dioxygenase from *Rhodococcus* sp. strain NCIMB12038. J Bacteriol. 1999;181:6200-6204.

86. Auffret MD, Yergeau E, Labbe D, Fayolle-Guichard F and Greer CW. Importance of *Rhodococcus* strains in a bacterial consortium degrading a mixture of hydrocarbons, gasoline, and diesel oil additives revealed by metatranscriptomic analysis. Appl Microbiol Biotechnol. 2015;99:2419-2430.

87. Brown LM, Gunasekera TS, Bowen LL and Ruiz ON. Draft Genome Sequence of *Rhodovulum* sp. Strain NI22, a Naphthalene-Degrading Marine Bacterium. Genome Announc. 2015;3.

88. Adriaenssens EM, Guerrero LD, Makhalanyane TP, Aislabie JM and Cowan DA. Draft Genome Sequence of the Aromatic Hydrocarbon-Degrading Bacterium *Sphingobium* sp. Strain Ant17, Isolated from Antarctic Soil. Genome Announc. 2014;2.

89. Dong C, Bai X, Lai Q, Xie Y, Chen X and Shao Z. Draft Genome Sequence of *Sphingobium* sp. Strain C100, a Polycyclic Aromatic Hydrocarbon-Degrading Bacterium from the Deep-Sea Sediment of the Arctic Ocean. Genome Announc. 2014;2.

90. Coppotelli BM, Ibarrolaza A, Dias RL, Del Panno MT, Berthe-Corti L and Morelli IS. Study of the degradation activity and the strategies to promote the bioavailability of phenanthrene by *Sphingomonas paucimobilis* strain 20006FA. Microb Ecol. 2010;59:266-276.

91. Schuler L, Jouanneau Y, Chadhain SM, Meyer C, Pouli M, Zylstra GJ, et al. Characterization of a ring-hydroxylating dioxygenase from phenanthrene-degrading *Sphingomonas* sp. strain LH128 able to oxidize benz[*a*]anthracene. Appl Microbiol Biotechnol. 2009;83:465-475.

92. Zhang R, Lai Q, Wang W, Li S and Shao Z. *Thioclava dalianensis* sp. nov., isolated from surface seawater. Int J Syst Evol Microbiol. 2013;63:2981-2985.
