# Supplementary material for: Polycyclic aromatic hydrocarbon (PAH) biodegradation capacity revealed by a genome-function relationship approach
Source: Environ Microbiome. 2023 Apr 30;18:39. doi: 10.1186/s40793-023-00497-7 (PMC10150532; doi:10.1186/s40793-023-00497-7)
Supplement: Supplementary file 3 — Additional file 3. Supplementary method and figures. [file 40793_2023_497_MOESM3_ESM.docx]

**Supplementary of**

Polycyclic aromatic hydrocarbon (PAH) biodegradation capacity revealed by a genome-function relationship approach

Yue Huang, Liguan Li, Xiaole Yin, Tong Zhang^*^

**Yue Huang:** Environmental Microbiome Engineering and Biotechnology Lab, Department of Civil Engineering, The University of Hong Kong, Pokfulam Road, Hong Kong, China. huangy00@hku.hk

**Liguan Li:** Environmental Microbiome Engineering and Biotechnology Lab, Department of Civil Engineering, The University of Hong Kong, Pokfulam Road, Hong Kong, China. liliguan@hku.hk

**Xiaole Yin:** Environmental Microbiome Engineering and Biotechnology Lab, Department of Civil Engineering, The University of Hong Kong, Pokfulam Road, Hong Kong, China. xiaole99@hku.hk

**Tong Zhang*** (Corresponding author). Environmental Microbiome Engineering and Biotechnology Lab, Department of Civil Engineering, The University of Hong Kong, Pokfulam Road, Hong Kong, China. [zhangt@hku.hk](mailto:zhangt@hku.hk); Tel.: +852-28578551; Fax:+852-25595337; ORCID ID: 0000-0003-1148-4322.

## Table of content

Supplementary methods

Figure S1. Numbers of reference protein sequences in PAH-degrading gene database.

Figure S2. Phylogenetic trees of *nah* gene cluster in the current database.

Figure S3. Phylogenetic trees of *nid* and *phd* gene cluster in the current database.

Figure S4. Phylogenetic trees of *nar* gene cluster in the current database.

Figure S5. Functional gene arrangements in known PAH-degrading strains.

Figure S6. The location of PAH-degrading genes in 47 known PAH-degrading bacterial strains.

Figure S7. Phylogenetic tree of 225 strains with a complete/near-complete PAH-degrading gene cluster.

Figure S8. The optimization of parameters in random forest analysis.

Figure S9. Variable importance contribution of different genes in terms of mean decrease accuracy and mean decrease gini in random forest analysis.

Figure S10. The PAH-degrading performance of six selected strains.

## Methods

## 1. Chemicals

Naphthalene (98% of purity) was purchased from Sigma-Aldrich (USA). Liquid chromatography/mass spectrometry grade acetonitrile was provided by Fisher Chemical (Pittsburgh, PA), and ultrapure water was produced by Barnstead EASY pure UV/UF water purification system. Six type strains, *Gordonia pthalatica* QH-11, *Leucobacter triazinivorans* JW-1, *Paraburkholderia fungorum* Croize P763-2, *Nocardioides humi* DCY24, *Microbacterium sediminis* YLB-01, and *Rhodococcus opacus* 1CP were purchased from DSMZ (German Collection of Microorganisms and Cell Cultures, German) to evaluate the predictions.

## 2. Degradation assay

The type strains were cultivated following the provided instructions. One single colony was inoculated into a 30 mL nutrition medium and incubated for 1~2 days at a suitable temperature. Then, 200 μL cell suspension was transferred to 30 mL fresh medium twice. The biomass was harvested by centrifugation at 4500 rpm for 20 min and washed thrice with mineral salts medium (MSM) medium. The cell pellets were resuspended in 200 mL MSM medium with 20 mg/L naphthalene as the sole carbon source (OD_595_ = 0.1). All the experiments were carried out in triplicates in 240 mL amber glass bottles (Boston round) with caps and magnetically stirred at room temperature. Then, a 0.5 mL sample was mixed with equal volume methanol and subsequently filtered with 0.22 µm polyvinylidene fluoride (PVDF) syringe filters (Millipore, German) to determine naphthalene concentration at designed intervals. The MSM with 20 mg/L naphthalene without inoculation was set as the control experiment. The degradation assay of each strain was conducted twice using independently grown colonies.

## 3. Analytical methods

Naphthalene was quantified by ultraperformance liquid chromatography (UPLC) system with fluorescence (FLR) detection (Acquity UPLC system, Waters). Instrument control and data collection were performed with MassLynx (v4.1) software (Waters). Sample (10 μL) was loaded into UPLC and separated by a BEH C18 column (2.1 × 50 mm, 1.7 µm) using binary gradient mobile phase at 50°C, i.e., water as mobile phase A and acetonitrile as mobile phase B. The gradient elution was performed as 50% A: 50% B for 2.5 min at a flow rate of 0.6 mL/min. The FLR detector was set with excitation and emission wavelengths of 280 and 324 nm, respectively. The quantification limit was 5 μg/L.

**Figure S1. Numbers of reference protein sequences in PAH-degrading gene database.** The original (seed) database was collected according to the PAH degradation pathways in the KEGG database, while the updated database was generated following the workflow described in the methods.

**Figure S2. Phylogenetic trees of *nah* gene cluster in the current database.** tr represents that the protein sequence is from the TrEMBL database, and sp indicates that the protein sequence is from the Swiss-Prot database.

**Figure S3. Phylogenetic trees of nid and *phd* gene cluster in the current database.** tr designates that the protein sequence is from the TrEMBL database, and sp means that the protein sequence is from the Swiss-Prot database.

**Figure S4. Phylogenetic trees of *nar* gene cluster in the current database.** tr means that the protein sequence is from the TrEMBL database, and sp represents that the protein sequence is from the Swiss-Prot database.

**Figure S5. Functional gene arrangements in known PAH-degrading strains.** The red tag represents the experimentally validated PAH-degrading bacteria, while the black tag denotes the strain with an identical functional gene arrangement and is phylogenetically related to PAH degraders.

**Figure S6. The location of PAH-degrading genes in 47 known PAH-degrading bacterial strains.**


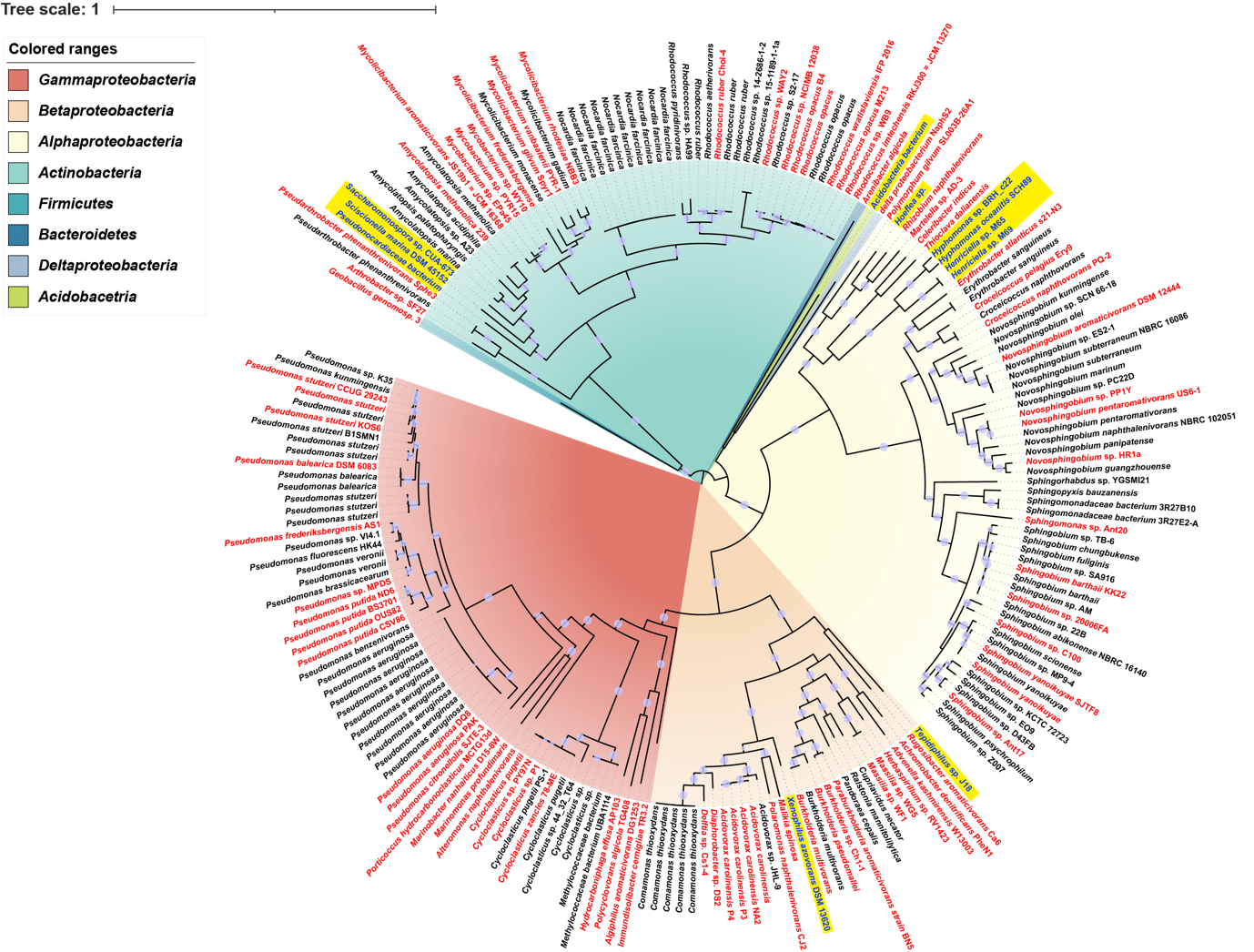


**Figure S7. Phylogenetic tree of 225 strains with a complete/near-complete PAH-degrading gene cluster.** The identified PAH degraders are shown in red font. The new PAH-degrading genera are highlighted in yellow shades. The tree was visualized in iTOL with the midpoint-rooting method, and only bootstrap values >70% were displayed.

**Figure S8. The optimization of parameters in random forest analysis.** (a) The variations of Matthew’s correlation coefficient (*MCC*) with the different True/False ratios in the dataset. Each ratio was estimated 1500 times with an independent random training dataset. (b) The optimization of tree numbers. (c) The bias of the random forest out-of-bag (OOB) errors under different m_try_ numbers from 1000 iterations.

**Figure S9. Variable importance contribution of different genes in terms of mean decrease accuracy and mean decrease gini in random forest analysis.**

**Figure S10. The PAH-degrading performance of six selected strains.** In these selected strains, two were predicted as PAH degraders based on random forest analysis. But none of them contained a complete PAH-catabolic pathway.
